# Supplementary material for: A Likelihood Approach for Real-Time Calibration of Stochastic Compartmental Epidemic Models
Source: PLoS Comput Biol. 2017 Jan 17;13(1):e1005257. doi: 10.1371/journal.pcbi.1005257 (PMC5240920; doi:10.1371/journal.pcbi.1005257)
Supplement: S1 File — (TAR.GZ) [file pcbi.1005257.s014.tar.gz › HSPH_Online-SI-Revision/PF08/n1-extreme/n1-extreme_PF_summary.pdf]

peak -8	R0	$1/\theta_2$	inf	inflog	newly	newlylog	Reff	AttackRate
mean	28.	31.9	522.8	0.7	1621.6	0.7	34.6	28.5
median	26.5	29.	308.8	0.6	313.1	0.6	33.	28.
Quantile .1	23.2	23.5	127.4	0.5	100.9	0.5	24.3	24.
Quantile .9	34.5	40.5	1197.2	0.9	1353.3	1.	47.4	32.
41	44	41						
length	50							

peak -4	R0	$1/\theta_2$	inf	inflog	newly	newlylog	Reff	AttackRate
mean	29.7	31.3	77.9	0.5	61.2	0.4	38.7	28.2
median	28.1	27.7	64.8	0.5	56.7	0.4	37.6	28.8
Quantile .1	22.9	23.9	54.2	0.3	49.3	0.3	29.5	20.
Quantile .9	37.2	44.2	111.7	0.6	79.9	0.5	48.8	32.9
34	46	39						
length	50							

peak	R0	$1/\theta_2$	inf	inflog	newly	newlylog	Reff	AttackRate
mean	26.1	30.	56.4	0.5	53.4	0.4	30.7	21.6
median	25.2	26.1	56.9	0.5	53.2	0.5	28.2	21.5
Quantile .1	21.2	20.9	48.2	0.3	44.4	0.3	24.3	16.5
Quantile .9	32.2	45.3	63.6	0.6	60.9	0.5	37.3	26.2
37	46	42						
length	50							

peak +4	R0	$1/\theta_2$	inf	inflog	newly	newlylog	Reff	AttackRate
mean	23.3	29.6	60.	0.4	48.	0.3	60.7	16.8
median	22.3	23.5	52.9	0.3	46.8	0.3	46.7	16.8
Quantile .1	19.3	19.4	42.8	0.2	36.1	0.2	25.9	10.7
Quantile .9	27.5	51.2	76.4	0.6	58.5	0.5	110.5	21.8
45	47	48						
length	50							

peak +8	R0	$1/\theta_2$	inf	inflog	newly	newlylog	Reff	AttackRate
mean	23.1	29.1	115.2	0.4	98.4	0.4	71.3	16.4
median	22.1	22.8	64.2	0.4	63.3	0.4	56.	15.8
Quantile .1	19.	19.3	47.4	0.3	47.6	0.2	29.9	11.
Quantile .9	27.7	51.9	255.1	0.6	200.2	0.5	139.3	20.9
45	47	50						
length	50							
